# JNK1 Phosphorylates SIRT1 and Promotes Its Enzymatic Activity

**DOI:** 10.1371/journal.pone.0008414

**Published:** 2009-12-22

**Authors:** Nargis Nasrin, Virendar K. Kaushik, Eric Fortier, Daniel Wall, Kevin J. Pearson, Rafael de Cabo, Laura Bordone

**Affiliations:** 1 Cardiovascular and Metabolism Disease Area, Novartis Institutes for BioMedical Research, Incorporated, Cambridge, Massachusetts, United States of America; 2 Analytical Sciences, Novartis Institutes for BioMedical Research, Incorporated, Cambridge, Massachusetts, United States of America; 3 Friedman School of Nutrition Science and Policy, Jean Mayer USDA Human Nutrition Research Center on Aging, Tufts University, Boston, Massachusetts, United States of America; 4 National Institutes of Health, National Institute on Aging, Bethesda, Maryland, United States of America; Duke University, United States of America

## Abstract

SIRT1 is a NAD-dependent deacetylase that regulates a variety of pathways including the stress protection pathway. SIRT1 deacetylates a number of protein substrates, including histones, FOXOs, PGC-1α, and p53, leading to cellular protection. We identified a functional interaction between cJUN N-terminal kinase (JNK1) and SIRT1 by coimmunoprecipitation of endogenous proteins. The interaction between JNK1 and SIRT1 was identified under conditions of oxidative stress and required activation of JNK1 *via* phosphorylation. Modulation of SIRT1 activity or protein levels using nicotinamide or RNAi did not modify JNK1 activity as measured by its ability to phosphorylate cJUN. In contrast, human SIRT1 was phosphorylated by JNK1 on three sites: Ser27, Ser47, and Thr530 and this phosphorylation of SIRT1 increased its nuclear localization and enzymatic activity. Surprisingly, JNK1 phosphorylation of SIRT1 showed substrate specificity resulting in deacetylation of histone H3, but not p53. These findings identify a mechanism for regulation of SIRT1 enzymatic activity in response to oxidative stress and shed new light on its role in the stress protection pathway.

## Introduction

Sirtuins are NAD^+^-dependent deacetylases (or ADP-ribosyltransferases) that remove acetyl groups from (or add ADP-ribose to) protein substrates, thereby regulating the biological function of their targets [1]. By catalyzing these reactions, sirtuins increase tissue and organism survival in a diversity of species, ranging from yeast to mammals [2], [3], [4]. The prototypical sirtuin, yeast Sir2p, is a NAD-dependent deacetylase that removes acetyl groups from histones to regulate chromatin structure [5]. In humans, the Sir2p homolog SIRT1 deacetylates transcription factors such as p53, FOXOs, and nuclear factor Kappa B (NFkB) mediating stress resistance, apoptosis, and inflammatory responses among other pathways[6]. Evidence from uni- and multicellular organisms indicates that SIRT1 has evolved to mediate signaling initiated by stressors, such as nutrient deprivation, to produce adaptation to enhance organism survival [7]. Consistent with this concept, extra copies of sirtuin genes increase survival in model organisms, including yeast, flies, and worms [2], [3], [4].

Recent studies have shown that SIRT1 can also protect organisms from the effects of oxidative stress [8], [9]. Reactive oxygen species (ROS) can damage a number of essential cellular components including lipid membranes, DNA, and proteins. The cellular response to an increase in ROS often involves the activation of numerous intracellular signaling cascades such as the family of mitogen-activated protein kinases (MAPKs) [10]. One family member of the MAPKs, c-Jun N-terminal kinase 1 (JNK1), plays a key role in signal transduction via growth factors, cytokines and cellular stresses such as heat shock, UV radiation and ROS. JNK1 phosphorylates components of the activator protein transcription factor complex such as c-Jun resulting in a change (e.g. hypertrophy or apoptosis) in cellular fate [11], [12], [13].

At present, the cellular machinery involved in the signal transduction from ROS to SIRT1 induced protection is unclear. Given the roles of JNK1 and SIRT1 in stress protection pathways, we tested for functional interactions between these proteins. In the present study, we report post-translational modification of SIRT1 as a result of JNK1 activation. JNK1 directly phosphorylates SIRT1 on three specific residues, concentrating it in the nucleus and resulting in selective activation of SIRT1, as measured by deacetylation of histone H3 (but not p53).

## Results

### Characterization of SIRT1:JNK1 Interaction

**Figure 1:**
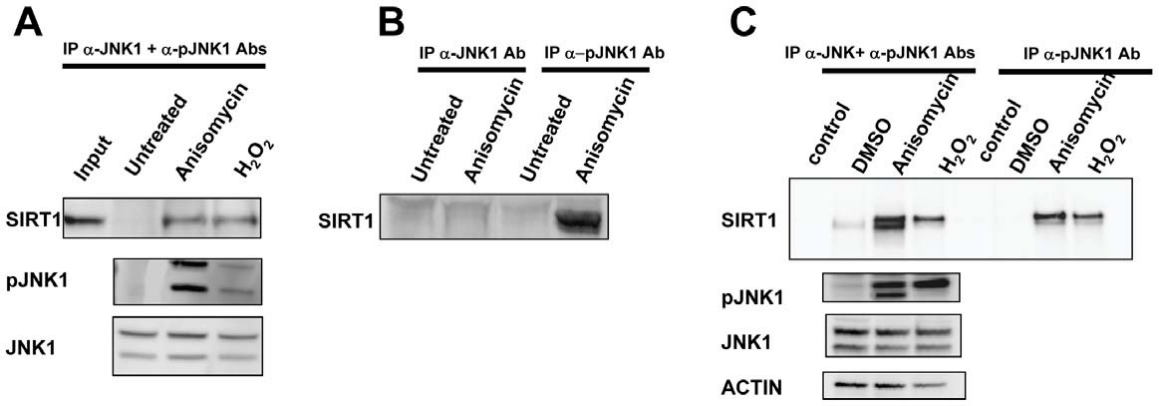
SIRT1 and JNK1 interact. *A*, Coimmunoprecipitation of SIRT1 with total JNK1 and phospho-JNK1 after treatment with anisomycin or H_2_O_2_. Anisomycin and H_2_O_2_ cause phosphorylation of JNK1 and interaction with SIRT1. *B*, Coimmunoprecipitation of SIRT1 with either total JNK1 or phospho-JNK1 from HEK293 cell lysates. SIRT1 immunoprecipitated specifically with phospho-JNK1 in response to anisomycin. *C*, Coimmunoprecipitation of SIRT1 with either total JNK1 plus phospho-JNK1 or phospho-JNK1 from C2C12 cell lysates. SIRT1 immunoprecipitated with phospho-JNK1 in response to anisomycin or H_2_O_2_.

SIRT1 has been implicated in a variety of cellular processes; however mechanisms regulating SIRT1 activity in cells are largely unknown. Given our interest in understanding SIRT1 regulation in the context of oxidative stress, we tested for a functional relationship between SIRT1 and JNK1 in a cellular system. To examine a potential interaction between SIRT1 and JNK1, co-immunoprecipitation experiments were performed from HEK293T cells. Both JNK1 and phosphorylated JNK1 (pJNK1) were immunoprecipitated and associated proteins were identified by Western blotting. An interaction between SIRT1 and JNK1 was observed only when cells were treated with the known JNK1 activators anisomycin or H_2_O_2_ (Fig. 1A). Anisomycin and H_2_O_2_treatment led to phosphorylation of JNK1, as expected. When either JNK1 or pJNK1 were immunoprecipitated following treatment of HEK293T cells with or without anisomycin, SIRT1 only co-immunoprecipitated with the antibody against pJNK1, further indicating SIRT1 interacts with phosphorylated- but not unphosphorylated JNK1 (Fig. 1B). A similar result was obtained using a different cell line (C2C12) where treatment with anisomycin or H_2_O_2_ resulted in interaction between SIRT1 and pJNK1 (Fig. 1C).

**Figure 2:**
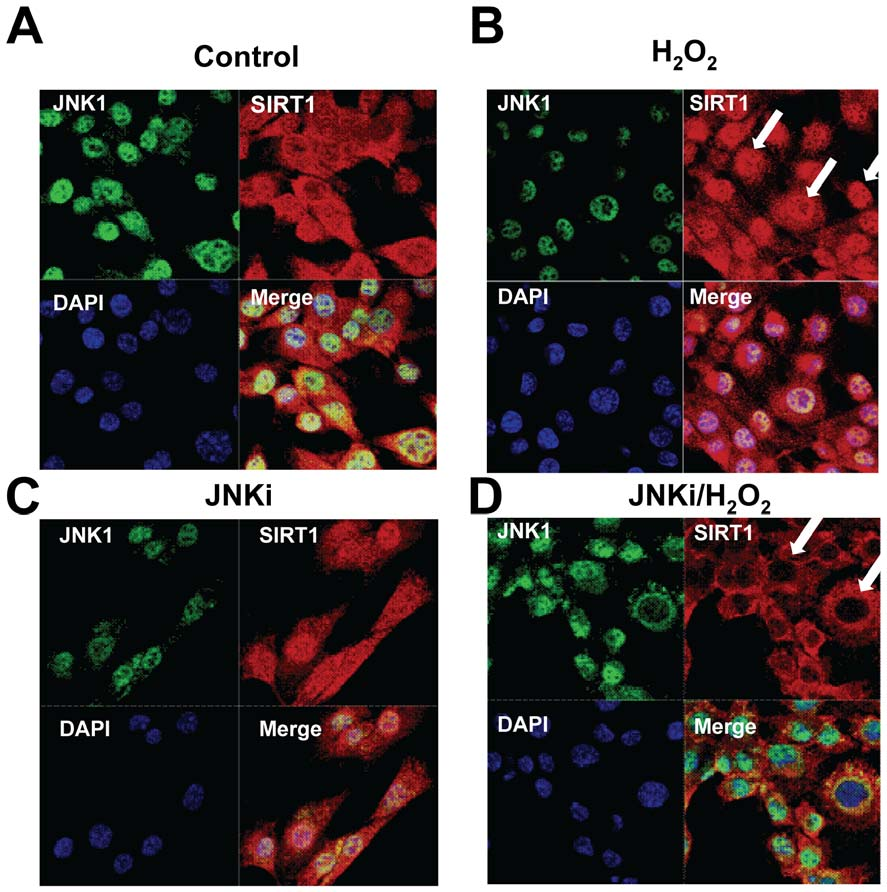
JNK1 regulates cellular localization of SIRT1. Immunohistochemistry of SIRT1 and JNK1. Cells were treated with control (*A*), H_2_O_2_ (*B*), JNKi (*C*) or H_2_O_2_ plus JNKi (*D*). Cellular localization of JNK1 was determined by anti-rabbit JNK antibody and SIRT1 by anti-mouse SIRT1 antibody. Cells were stained with DAPI stain to visualize nuclei. Arrows show SIRT1 localization.

To determine the mechanism of regulation of SIRT1 by JNK1, cellular localization of SIRT1 was examined after treatment with H_2_O_2_ in the presence or absence of JNKi, a cell permeable inhibitor of JNK1. Immunohistochemistry demonstrated SIRT1 was localized both in cytoplasmic and nuclear compartments in control C2C12 cells (Fig. 2A). Treatment with H_2_O_2_ increased nuclear localization of SIRT1 (Fig. 2B), and inhibition of JNK1 by JNKi resulted in decreased nuclear localization of SIRT1 in the presence of H_2_O_2_ (Fig. 2D). JNKi alone did not show any effect (Fig. 2C). JNK1 was primarily localized to the nucleus. These findings demonstrate that JNK1 may play a role in the function and localization of SIRT1.

### SIRT1 Does Not Affect JNK1 Activity

**Figure 3:**
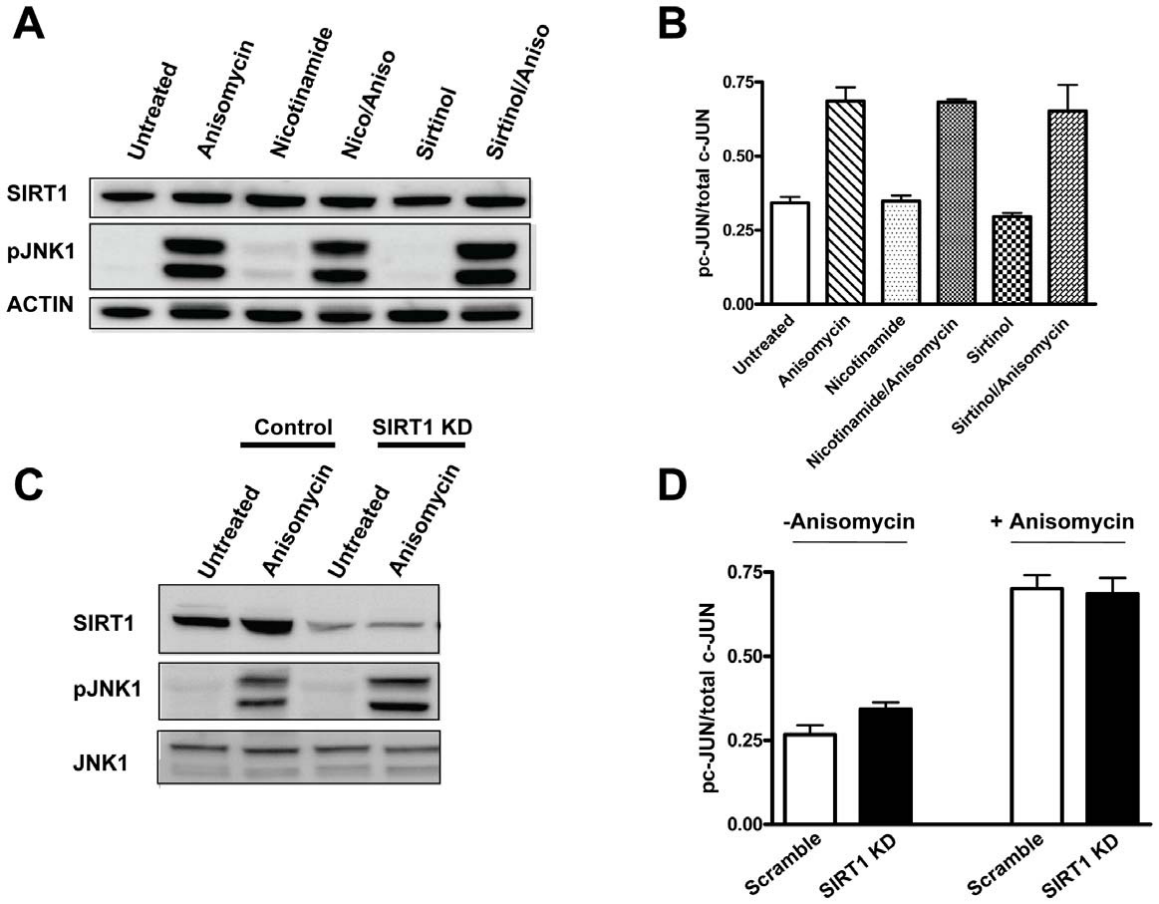
SIRT1 does not regulate JNK1 phosphorylation or activity. *A*, Inhibition of SIRT1 activity by nicotinamide or Sirtinol does not affect phosphorylation of JNK1 in response to anisomycin. *B*, Inhibition of SIRT1 activity by nicotinamide or Sirtinol does not affect activity of JNK1 in response to anisomycin. JNK1 activity was measured by its ability to phosphorylate c-Jun. *C*, Knockdown of SIRT1 by siRNA does not affect anisomycin-mediated phosphorylation of JNK1. *D*, Knockdown of SIRT1 does not affect anisomycin-stimulated activity of JNK1. Clear bars are control. Black bars are SIRT1 knockdown.

Next, we determined that the interaction between SIRT1 and JNK1 has no effect on JNK1 activity. HEK293T cells were treated with anisomycin and SIRT1 was inhibited acutely (nicotinamide/Sirtinol) or chronically (knockdown by siRNA). Acute inhibition of SIRT1 activity did not significantly change the phosphorylation or activity of JNK1 by anisomycin treatment (Fig. 3A, 3B). In addition, chronic inhibition via knockdown of SIRT1 did not significantly change the phosphorylation state or activity of JNK1 in HEK293T cells treated with or without anisomycin (Fig. 3C, 3D). Thus, SIRT1 does not significantly affect JNK1 phosphorylation or activity under these conditions.

### JNK1 Directly Phosphorylates SIRT1

**Figure 4:**
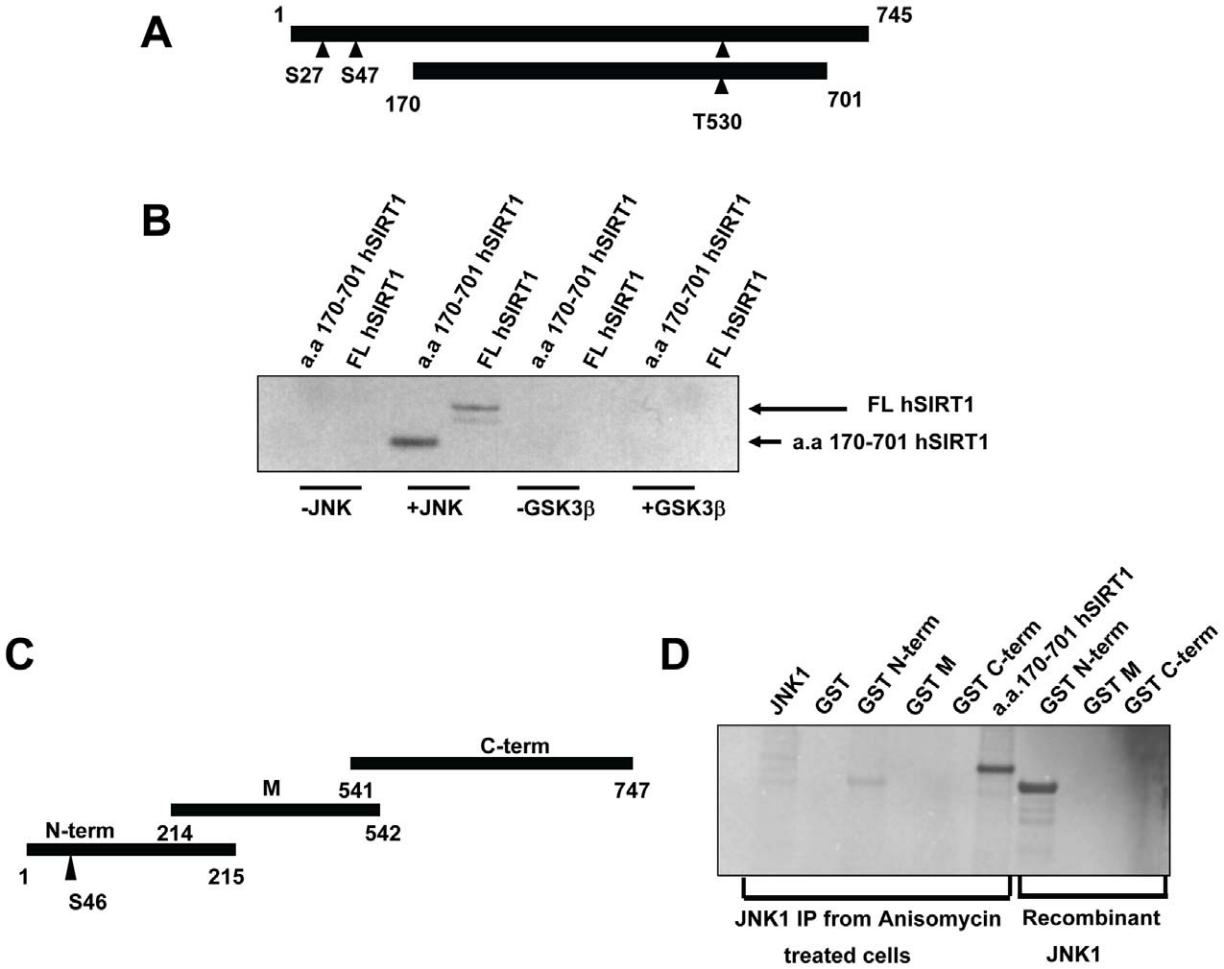
SIRT1 is phosphorylated by JNK1. *A*, Schematic representation of full length human SIRT1 (FL hSIRT1) protein and a truncated form (aa 170–701 hSIRT1). Triangles show putative JNK1 phosphorylation sites. *B*, phosphorylation of SIRT1 by recombinant JNK1. FL hSIRT1 or a.a. 170–701 hSIRT1 was incubated with recombinant JNK1 or GSK3β in the presence of γ-[^32^P]-ATP. *C*, Schematic representation of mouse SIRT1 GST fragments. Triangle represents the putative JNK phosphorylation site. *D, In vitro* phosphorylation of mouse SIRT1 GST when treated with JNK1 immunoprecipitates from anisomycin-treated C2C12 cells or recombinant JNK1. Truncated hSIRT1 (a.a. 170–701 hSIRT1) was used as positive control.

**Figure 5:**
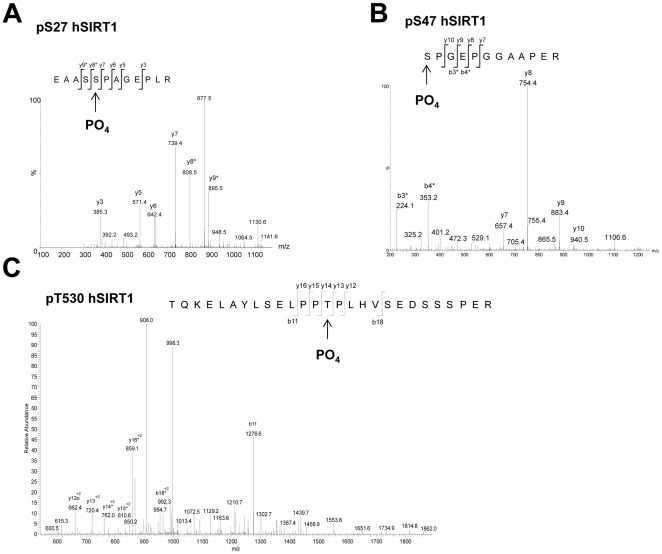
Phosphorylation of hSIRT1. *A* and *B*, These peptide MSMS fragmentation spectra were obtained from the *ex vivo* FL hSIRT1 IP samples after SDS PAGE and in-gel digestion. The spectrum in *A* shows phosphorylation of pS27 with neutral loss of 98 Da from the y8*, y9* and y10* ions (indicated with a *). Spectrum *B* shows phosphorylation of pS47 with neutral loss of 98 Da from the b3* and b4* ions, but no neutral loss from the y ions. *C*, The MSMS spectrum shown here was derived from *in vitro* experiments with hSIRT1 (a.a. 170–701) and shows phosphorylation of pT530 with neutral loss of 98 Da for y14* to y17*. These spectra were produced from Mascot database searches with high confidence scores using data generated from a nanoLC LTQ FT mass spectrometer.

To determine if JNK1 phosphorylated SIRT1 directly, we measured radioactive phosphate incorporation into SIRT1 by *in vitro* kinase assays and autoradiography with JNK1, or a non-specific kinase, namely glycogen synthase kinase-3β (GSK3β). Human SIRT1 contains two previously identified phosphorylation sites (Ser27 and Ser47) of unknown function. Recombinant purified hSIRT1 (FL hSIRT1) protein was phosphorylated by JNK1 but not GSK3β (Fig. 4B). These findings were corroborated using SIRT1 immunoprecipitated from HEK293 cells treated with anisomycin (data not shown). Interestingly, a truncated hSIRT1 protein construct (SIRT1 a.a.170–701) not containing the Ser27 and Ser47 phosphorylation sites, was also phosphorylated by JNK1, (but not GSK3β) indicating a potential novel site. Therefore, we mapped JNK1 phosphorylation sites on SIRT1 using mass spectrometry (Fig. 5). Three phosphorylation sites were identified; the previously known Ser27 and Ser47 sites and a novel site, Thr530. JNK2 and JNK3 also phosphorylated FL hSIRT1 (data not shown), albeit to a lesser degree, (relative to JNK protein levels) indicating that phosphorylation of SIRT1 by JNK proteins may represent a general mechanism for SIRT1 activity regulation.

To further assess the specificity of JNK1 phosphorylation of SIRT1, we used recombinant mouse SIRT1 (mSIRT1). In contrast to hSIRT1, mSIRT1 does not contain the Ser27 and Thr530 phosphorylation sites (Fig. 4C). An N-terminal fragment of mSIRT1 (N-term) was effectively phosphorylated by recombinant JNK1 or by JNK1 immunoprecipitated from anisomycin-treated HEK293T cells (Fig. 3D), whereas a C-terminal fragment of mSIRT1 was not (Fig. 4D). These results demonstrate that JNK1 is capable of phosphorylating both human and mouse SIRT1 protein, but differentially based on their amino acid sequences. Given that both the mouse and human SIRT1 proteins contain many potential JNK1 phosphorylation sites (based on the S/T-P JNK1 phosphorylation motif), the results also suggest a degree of phosphorylation specificity.

### Mutation of JNK1 Phosphorylation Sites on SIRT1 Abrogates Functional Response

**Figure 6:**
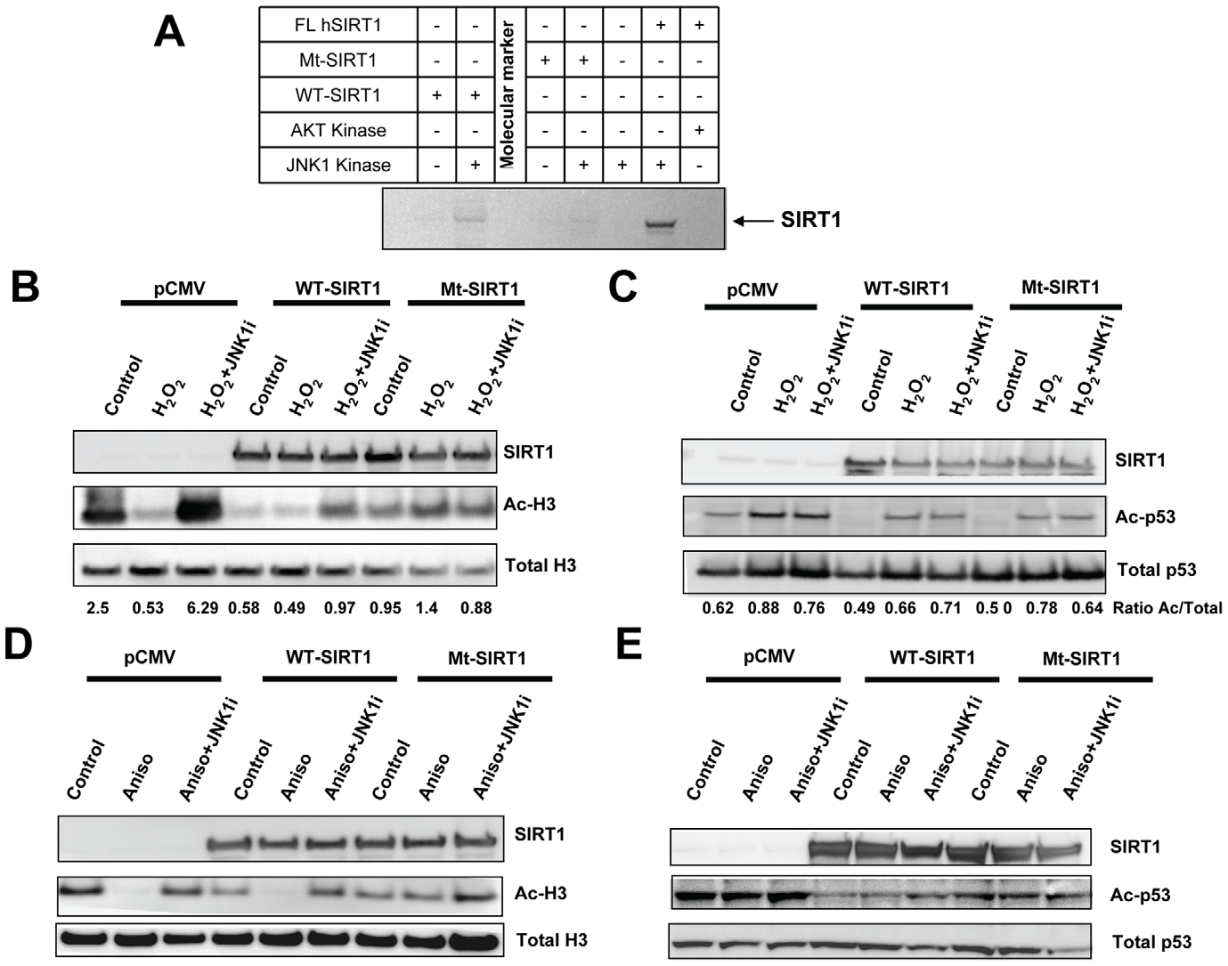
Mutation of putative JNK phosphorylation sites on hSIRT1 reduces phosphorylation and activity of SIRT1. *A*, *In vitro* phosphorylation of full length (FL) hSIRT1 or SIRT1 with the putative JNK phosphorylation sites mutated (Mt-SIRT1). FL hSIRT1 or Mt-SIRT1 was incubated with recombinant JNK1. *B*, Mt-SIRT1 shows decreased activity in deacetylation of histone H3. Cells were transfected with the empty vector (CMV), WT hSIRT1 (WT) or Mt-SIRT1 (Mt) then treated with H_2_O_2_ or H_2_O_2_ plus JNK inhibitor (JNKi). SIRT1, acetylated histone H3 (Ac-H3) or total histone H3 were examined by Western blotting. *C*, Mt-SIRT1 shows similar activity to WT SIRT1 in deacetylation of p53. Experiment performed as in *B*, except that acetylated p53 (Ac-p53) and total p53 levels were monitored. The ratio Ac/Total shows the quantification of the expression levels of the acetylated substrates *vs*. the total levels of the same substrates. *D*, Mt-SIRT1 shows decreased activity in deacetylation of histone3 (H3). Cells were transfected with the empty vector (CMV), WT hSIRT1 (WT) or Mt-SIRT1 (Mt) then treated with anisomycin or anisomycin plus JNK inhibitor (JNKi). SIRT1, acetylated histone H3 or total H3 were examined by Western blotting. *E*, Mt-SIRT1 shows similar activity to WT SIRT1 in deacetylation of p53 when cells were treated with anisomycin. The experiment was performed as in *D*, and the levels of acetylated p53 (Ac-p53) and total p53 were determined by Western blotting.

To probe the function of SIRT1 phosphorylation, a mutant hSIRT1 (Mt-SIRT1) protein was generated by site-directed mutagenesis of Ser27, Ser47 and Thr530 to alanine residues. Whereas recombinant FL hSIRT1 was effectively phosphorylated by JNK1 *in vitro*, phosphorylation of Mt-SIRT1 by JNK1 was dramatically reduced (Fig. 6A). To assess the functional relevance of these phosphorylation sites, WT or Mt-SIRT1 were expressed in HEK293T cells and the activity of SIRT1 was monitored by testing the acetylation status of two SIRT1 targets. As expected, cells transfected with WT-hSIRT1 show markedly decreased acetylation of the SIRT1 substrates histone H3 and p53 (Fig. 6B and 6C). Cells transfected with Mt-SIRT1 had differential effects, depending on the SIRT1 protein substrate measured. There was not a significant difference in p53 acetylation in cells transfected with either WT-hSIRT or Mt-SIRT1 when treated with H_2_O_2_ (Fig. 6C), indicating no dependence on SIRT1 phosphorylation state. In contrast, expression of Mt-SIRT1 caused only a modest decrease in acetylated H3 (Ac-H3) demonstrating substrate specificity for this response (Fig 6B). In addition, treatment of cells with H_2_O_2_ resulted in a significant decrease in Ac-H3 and this effect was blocked by addition of JNKi (Fig. 6B). Similarly, JNKi reduced the ability of over-expressed WT-SIRT1 to deacetylate H3. Treatment with H_2_O_2_ did not decrease Ac-H3 levels in cells expressing the Mt-SIRT1 (Fig. 6B) demonstrating H_2_O_2_-mediated deacetylation of histone H3 is dependent on phosphorylation of SIRT1. On the contrary, treatment with H_2_O_2_ increased acetylated p53 (Ac-p53) levels even in cells over-expressing SIRT1 (either WT- or Mt-SIRT1) (Fig. 6C). Similar results were obtained when cells were treated with anisomycin as shown in Figure 6D and E. These results demonstrate substrate specificity for the ability of JNK1 to modulate SIRT1-mediated deacetylation.

## Discussion

In this paper we describe a novel mechanism whereby oxidative stress, via JNK1, regulates SIRT1 activity by altering its subcellular localization and activity. First, we demonstrated that SIRT1 and JNK1 functionally interact, but only when JNK1 is activated by anisomycin or H_2_O_2_. Second, we showed that SIRT1 localization changes after treatment with H_2_O_2_, an effect blocked by treatment of cells with a JNK1 inhibitor. Third, we confirmed JNK1 as a kinase involved in phosphorylating SIRT1 at previously identified sites (Ser27 and Ser47), and identified a novel JNK1 phosphorylation site (Thr530) on SIRT1. Fourth, we demonstrated a functional role of JNK1 phosphorylation of SIRT1, selective deacetylation of histone H3 but not another SIRT1 substrate, namely p53. Mutations of these sites into alanines abolished JNK1-mediated phosphorylation of SIRT1 and abrogated the functional changes seen with respect to histone H3. Finally, we determined that the SIRT1:JNK1 interaction did not lead to a change in JNK1 activity, as measured by levels of phosphorylated cJun.

The findings presented here provide a molecular mechanism for activation of SIRT1 in response to oxidative stress. This is the first report identifying JNK1 as a kinase that functionally modifies SIRT1, increasing SIRT1 activity and altering SIRT1 localization. Specifically, phosphorylation of SIRT1 increases deacetylation of histone H3. By modifying chromatin SIRT1 induction might modulate a set of genes that will confer protection from oxidative stress. In support of this hypothesis, Berthiaume and colleagues [14] have shown that high levels of cellular oxidative stress globally inhibit gene transcription and this correlates with a significant decrease in histone H3 and H4 acetylation. This could be a protective mechanism to promote cell survival. Cells may induce histone deacetylation to increase DNA integrity and reduce DNA damage mediated by free radicals. Further studies to identify changes in specific genes related to deacetylation of histone H3 are ongoing. The findings presented here have implications in understanding how SIRT1 might mediate the survival of cells under oxidative stress conditions.

Recently, it has been shown that JNK2 can phopshorylate SIRT1 at Ser27 under basal conditions, increasing SIRT1 protein stability [15]. We can not rule the possibility that JNK2 may play a role in H_2_O_2_ mediated phosphorylation of SIRT1. In addition, cyclin dependent kinases (Cyclin B/Cdk1) phosphorylate SIRT1, increasing deacetylase activity and regulating cell cycle progression [16]. These studies suggest hierarchical levels of phosphorylation as a second-site on SIRT1 must be phosphorylated to detect Cyclin B/Cdk1 regulation. These findings, coupled with the work presented here shed new light on how SIRT1 can be modified in cells by postranslational modifications and in particular how SIRT1 can differentially respond to physiological events, like oxidative stress.

SIRT1 is a cell survival factor that may increase resistance to oxidative stress in mammalian cells. In normal human fibroblast IMR-90 cells, H_2_O_2_-induced apoptosis was attenuated by SIRT1 overexpression [17]. In HEK293T cells treated with serum collected from calorie-restricted rats, SIRT1 expression level was increased and Bax-mediated apoptosis was inhibited [18]. More recently, it was shown that SIRT1 plays an essential role in mediating cell survival in cardiac myocytes [19], [20]. Kume et al [8] have shown that H_2_O_2_-induction of p53 acetylation was decreased in mesangial cells overexpressing SIRT1, similar to data we obtained in a different cell line (HEK293T). In addition, neither overexpression nor knockdown of SIRT1 affected H_2_O_2_-mediated phosphorylation of MAP kinases (p38, JNK or MAPK). This is in agreement with our finding that SIRT1 has no effect on JNK1 activity. Tanno et al [20] indicate that SIRT1 nucleocytoplasmic shuttling, in response to oxidative stress, is a regulatory mechanism which may increase cellular resistance to apoptosis. In keeping with this notion, our data suggest that phosphorylation via JNK1 is an important regulator of SIRT1 subcellular localization and function. Taken together, these studies support the notion that phosphorylation of SIRT1 by JNK1 is a protective mechanism against oxidative stress.

The cellular balance between SIRT1 and poly(ADP-ribose) polymerase-1 (PARP-1) activity may determine the effects of oxidative stress on cell survival. In contrast to the present study, activation of JNK1 (via oxidative stress) has previously been shown to mediate cell death via PARP-1 activation [21]. PARP-1 is a member of a nuclear enzyme family that functions as post-translational regulators of DNA damage repair. Despite its beneficial effect in DNA repair, PARP-1 activation has been implicated in necrotic cell death. This apparent contrast in the regulation of the stress response by JNK1 may be a function of the level of cellular damage. In the present study cells were treated with H_2_O_2_ for 1 h, resulting in minimal cell death, whereas the JNK1:PARP-1 association required longer (∼6 h) cellular H_2_O_2_ exposure. Perhaps during prolonged H_2_O_2_ exposure, DNA damage accumulates and massive PARP-1 activation quickly depletes cellular NAD^+^ (a SIRT1 co-substrate), leading to decreased SIRT1 activity and necrotic cell death. Conversely, acute (or low-level) exposure to oxidative stress activates SIRT1, promoting the chance for cellular recovery.

During the last two decades progress in the genetics of aging in invertebrate models such as *C. elegans* and *D. melanogaster* has clearly demonstrated the existence of regulatory pathways that control the rate of aging in these organisms, such as the insulin-like pathway, the JNK pathway and the Sir2 deacetylase pathway [22]. It has been shown both in *Drosophila* and in *C.elegans* that activation of the stress responsive JNK pathway has major implications for modulating lifespan. In both organisms it seems that the effect of overexpression of JNK on increased lifespan was mediated by nuclear localization of dFoxo/Daf16 and induction of its target genes [23], [24]. Here we show that in mammalian systems an additional pathway might be responsible to protect cells under oxidative stress conditions. It is not surprising that both overexpression of SIRT1 and/or activation of the JNK pathway lead to accumulation of less oxidative damage and increase lifespan. The survival-enhancing characteristics of sirtuins suggest that they influence mammalian responses to toxicity and could be new therapeutic targets for improvement of tissue survival in toxicity-related diseases, particularly neurodegenerative disorders.

In conclusion, oxidative stress activates JNK1, which directly phosphorylates SIRT1 at Ser27, Ser47, and Thr530, concentrating it into the nucleus. In the nucleus, phosphorylated SIRT1 selectively deacetylates histone H3, but not p53. These findings establish a molecular mechanism linking oxidative stress to SIRT1.

## Materials and Methods

### Cell Culture

Human embryonic kidney (293T) and mouse myoblast (C2C12) cells from ATCC were cultured in Dulbecco's modified Eagle medium (DMEM). The media was supplemented with 10% fetal bovine serum (FBS) and 10 mM HEPES. The day before transfection, 293T cells at 3×10^5^ density were plated onto a 10 cm dish. For overexpression of SIRT1, 10 µg of wild-type and mutant plasmids were used for each dish. The cells were harvested at 72 hours post-transfection. For knockdown (KD) of SIRT1, SiRNAs targeting the SIRT1 and control siRNA were used at 20 nM concentration.

### SIRT1 Plasmid Construction

The SIRT1 phospho-mutant construct was subcloned into the pCMV Tag 4A (Flag tag) vector from Stratagene. Ser27Ala, Ser47Ala and Thr530Ala mutations were performed by using site-directed mutagenesis. The Ser27Ala mutagenesis was performed by using the Advantage-GC 2 polymerase kit. The GC-melt concentration was used from 0–1.5 M as recommended. PCR was performed at 94°C for 3 min, then 30 cycles of 94°C for 30 sec and 68°C for 6 min and one additional 68°C incubation for 6 min. A DpnI restriction digest was used to eliminate parental DNA stands and the mixture was transformed using Stratagene XL1-Blue Supercompetent cells. In addition a PstI silent restriction site was incorporated into the mutant primers for candidate screening. The Ser47Ala, Thr530Ala and stop mutation sites were mutated by using the QuikChange II XL Site-Directed Mutagenesis Kit. All mutations were sequence verified and primers are listed in Table S1. Base pairs in bold represent the mutated sites.

### Treatments

To activate JNK1 activity, cells were treated with 500 µM (13.3 M stock supplied) H_2_O_2_ for 30 min or with 10 µg per ml (10 mg ml^−1^ stock in distilled water) anisomycin for 30 min at 37°C. To block the JNK1 kinase activation, cells were treated with 1 µM cell-permeable JNK inhibitor 1, (EMD Chemicals, Inc., Gibbstown, NJ, USA) overnight at 37°C before being exposed to anisomycin for 30 min. Cells were treated with 10 mM nicotinamide (1 M stock solution in distilled water) or 50 µM Sirtinol (50 mM stock in DMSO) overnight before treatment with H_2_O_2_ or anisomycin. Controls for the experiments were treated with distilled water and/or DMSO. For detection of acetylated Histone 3 (H3), the cells were incubated overnight with Trichostatin A (TSA) at 400 nM concentration.

### Antibodies

The rabbit anti-SIR2 polyclonal antibody was purchased from Millipore (Billerica, MA, USA). Rabbit anti JNK antibody, rabbit anti-phospho JNK pAb were purchased from Cell Signaling Technology (Danvers, MA, USA). For immunofluorescence, anti-mouse JNK1 mAb was purchased from BD Bioscience (San Jose, CA, USA). Rabbit anti-Histone H3, Acetylated (1–20), pAb was purchased from EMD Chemicals, Inc. Rabbit anti-Histone H3 pAb was purchased from Cell Signaling Technology.

### Western Blotting and Co-Immunoprecipitation

For Western blot analysis, cells were lysed in RIPA lysis buffer supplemented with 10 mM NaF, 2 mM Na_3_VO_4_, 1 mM PMSF, 5 µM pepstatin, 1 mM DTT, 10 U/ml aprotinin, 0.5 mM Na-Pyrophosphate and 1 complete mini protease inhibitor cocktail tablet per 10 ml. The lysate was incubated on ice for 30 min followed by centrifugation at 14,000 rpm for 20 min at 4°C. The protein concentration was determined using the Coomassie plus protein assay reagent. Fifty µg lysates were denatured by adding SDS loading buffer and heated at 99°C for 10 min. The denatured samples were size fractionated by 4–12% SDS-PAGE and transferred onto a 0.4 µ PVDF membrane. Primary antibodies (listed below) at 1∶1000 dilutions were used overnight at 4°C on a platform shaker. After incubation for 1 h with the secondary antibody (donkey anti-rabbit HRP conjugated, 1∶5000 or sheep anti-mouse HRP conjugated), the signals were detected by ECL enhanced chemiluminescence reagent using Fujifilm scanner.

For co-immunoprecipitation assays, protein A-Sepharose beads were equilibrated with RIPA buffer and pre-coated with BSA (0.5% for 1 hour). Immunoprecipitation assays were then performed using 500 to 1,200 µg of total protein. Lysates were pre-cleared by the addition of 30 µl of 50% slurry of pre-coated protein A-sepharose beads per 500 µg of protein, by rocking at 4°C for 1 h. The supernatant was collected by centrifugation and the SIRT1 protein was immunoprecipitated with rabbit anti-mouse SIR2α polyclonal antibody (5 µl per 1000 µg of protein) from Upstate (Millipore, Billerica MA, USA) by rocking overnight at 4°C. Fifty to 100 µl of 50% slurry (pre-coated protein A Sepharose beads) were added to the mixture and incubated for 2 h at 4°C with rocking. The beads were washed three times with RIPA buffer containing protease and phosphatase inhibitors. The immunoprecipitates were eluted by boiling for 5 min in SDS sample buffer and subjected to SDS-PAGE analysis and Western blotting as described above with SIRT1 and JNK1 antibodies.

### Immunofluorescence

C2C12 cells were cultured as described above. The cells were plated on a Mat-Tek 35 mm glass bottom dish for H_2_O_2_ and/or JNK inhibitor treatment. After treatment, the cells were washed twice in PBS and fixed in 4% (in distilled water) para-formaldehyde fixative for 30 min; they were then washed and permeabilized three times (10 min each time) with 0.5% Triton-X-100 in PBS. For immunostaining on fixed cells, the dishes were rinsed with PBS containing 0.05% Tween-20 (PBST) and incubated in blocking reagent (1% BSA, 20% horse serum) for 1 h at room temperature. The next stage involved overnight incubation at 4°C in a 1∶50 dilution of rabbit anti-SIR2 and mouse anti-JNK1 antibodies followed by three washes with PBST before incubation in Alexa-488-conjugated goat anti-rabbit or donkey anti-mouse antibody at a dilution of 1∶2000 for 1 h in dark conditions. The cells were then washed three times in PBST and once in PBS before being exposed to 300 nM DAPI and examined in a Zeiss LSM 510 laser-scanning confocal microscope.

### JNK1 Activity

PathScan phospho-c-Jun (Ser63) and total c-Jun sandwich ELISA kit were used for measuring indirect JNK1 activity. Cell extracts from control and SIRT1 knockdown were used for measuring phosphorylation of c-Jun kinase. The ratio of phospho c-JUN over total c-JUN was used to measure the level of activation.

### SIRT1 Recombinant Protein Purification

For *in vitro* kinase studies, recombinant full-length human SIRT1 (FL hSIRT1) was purchased from Biomol International, LP (Plymouth Meeting, PA). Truncated SIRT1 (amino acids 170–701) was obtained by subcloning poly-histidine, S-tagged SIRT1 into a pNAT40 vector containing a PreScission protease cleavage site after the S-tag. The resulting construct was transformed into BL21DE3 cells, which were grown to an A600 of 0.6–0.8 prior to induction with isopropyl-β-D-thiogalactopyranoside (IPTG) for 6 h. Cells were harvested and stored at −80°C until used. The histidine, S-tagged SIRT1 protein was isolated using affinity, anion exchange, and size exclusion chromatography. Briefly, cells were lysed (using a French pressure cell) in lysis buffer containing 50 mM Tris pH 8, 300 mM NaCl, 2 mM TCEP, and Complete protease inhibitor cocktail tablets (Roche Applied Science, Indianapolis, IN). Cell debris was removed by centrifugation (29,000 xg, 1 h, 4°C). The supernatant was loaded onto a nickel column (HisPrep FF 16/10, GE Healthcare, Piscataway, NJ), washed with lysis buffer, and eluted using an imidazole gradient (0–500 mM). SIRT1 fractions were pooled and subject to PreScission protease (GE Healthcare) treatment per manufacturer's instructions to remove affinity tag. The cleaved product was dialyzed against lysis buffer containing 50 mM NaCl, loaded onto a mono Q column (10/100 GL, GE Healthcare) and eluted using a NaCl gradient (50–1000 mM). SIRT1 fractions were pooled, loaded onto a Superdex-200 column, and eluted in 50 mM Tris pH 8, 500 mM NaCl, 2 mM TCEP, and 5% glycerol. SIRT1 fractions were pooled at stored at −80°C until used. The mouse GST-SIRT1 fusion proteins were obtained from Department of Diabetes, Mass General Hospital, Massachusetts under MTA contract (Maria Alexander Bridges).

*In vitro kinase assay*-Equal amounts (1 µg) of the FL SIRT1 and GST-SIRT1 fusion proteins were incubated with the activated kinase (10 U) in kinase-specific reaction buffer containing 10 µCi (0.37 MBq) Easytide γ-33P–adenosine triphosphate (NEG602H). The kinases used for these assays were JNK1 and GSK3β kinase. The kinase reactions were incubated in kinase buffer at 30°C for 30 minutes and then stopped by the addition of Laemmli sample buffer. The reactions were boiled and separated by SDS-PAGE. The gels were dried and protein was detected by autoradiography. For detecting the phosphorylation status of wild-type and mutant SIRT1 proteins, both wild-type and mutant constructs were overexpressed in 293T cells along with their control plasmid. The equal amount of overexpressed proteins was immunoprecipitated using rabbit anti SIR2 and sepharose Protein A beads. The pulled down proteins were washed 3x times with kinase buffer. After washing, the proteins bound to beads, were resuspended with 20 µl kinase buffer and kinased with recombinant JNK1.

### Mass Spectrometry

In a typical experiment, endogenous SIRT1 was immunoprecipitated from 293T cells using the rabbit anti-SIR2 antibody. The complex was run on a SDS denaturing gel. After CBB staining and destaining the gel, gel bands were excised, washed using water/acetonitrile (1/1) with 25 mM ammonium bicarbonate and vortexing for 20 minutes. After dehydrating the bands, the proteins therein were reduced using 10 mM DTT for 45 minutes at 58°C. Next the proteins were alkylated on the cysteines using iodoacetamide at room temperature, in the dark. The gel pieces were then dried and rehydrated using the tryptic, chymotryptic or GluC enzyme solution (1/50, enzyme/protein) in 50 mM ammonium bicarbonate. Solution based samples, such as those from the *in vitro* studies, were prepared in similar fashion, but with no need to extract peptides from the gel matrix. The samples were digested overnight at 37°C for trypsin and room temperature for chymotrypsin and GluC. Peptides from the in-gel digests were extracted using 50% acetonitrile, 45% water and 5% formic acid with vortexing. After proteolysis peptides were dried down and rehydrated in a small volume for loading to the liquid chromatography mass spectrometry (LC/MS) instrument. Liquid chromatography tandem mass spectrometry (LC/MS/MS) was performed using a nano-flow RP HPLC (LC Packings Nanomate, Dionex) flowing at 250 nL/min through a 10 cm×75 µM capillary column (Picotip, New Objective) using silica based C18 particles (3 µM diameter and 100 angstrom pore size, Magic C18, Michrom Bioresources, Inc.). The gradient was over 55 minutes from 5 to 35% acetonitrile in 0.1% formic acid. Mass spectrometry of eluting peptides was achieved using either a Q-Tof Global (Micromass Q-Tof Global Ultima, Waters) or LTQ FT (Thermo) set to acquire in data dependent mode. Processed data was analyzed using Mascot (Matrix Sciences) to identify the proteins from the gel bands and sites of phosphorylation on detected peptides. The reference databases used were the human/mouse/rat RefSeq protein database as well as a small custom protein database.

## Supporting Information

Table S1 The primers used to create the mutant SIRT1 are described in this table.
(0.03 MB DOC)
