# Supplementary material for: JNK1 Phosphorylates SIRT1 and Promotes Its Enzymatic Activity
Source: PLoS One. 2009 Dec 22;4(12):e8414. doi: 10.1371/journal.pone.0008414 (PMC2793009; doi:10.1371/journal.pone.0008414)
Supplement: Table S1 — The primers used to create the mutant SIRT1 are described in this table. (0.03 MB DOC) [file pone.0008414.s001.doc]

| **Primers** | **Sequence** |
| --- | --- |
| Ser27Ala forward | 5’CGACAGGGAGGCCGCGTC**GG**CCCCTGCAGGGGAGCCGCTCCGCAAGAGG 3’ |
| Ser47Ala forward | 5’CGGCCTCGAGCG**GG**CCCCGGGCGAGCCC 3’ |
| Thr530Ala forward | 5’CAGAGTTGCCACCC**GAC**CCTCTTCATGTTTC 3’ |
| Stop codon to Ala forward | 5’ CCATCAAACAAATCA**GCG**GTCGACCTCGAGG 3’ |
